# Intra-serotypic antigenic diversity of dengue virus serotype 3 in Thailand during 2004–2015

**DOI:** 10.1017/S0950268823001991

**Published:** 2024-01-08

**Authors:** Promsin Masrinoul, Panumas Sun-Arlee, Sutee Yoksan, Duangnapa Wanlayaporn, Sanjira Juntarapornchai, Surat Punyahathaikul, Kunjimas Ketsuwan, Somnuek Palabodeewat, Alita Kongchanagul, Prasert Auewarakul

**Affiliations:** 1 Center for Vaccine Development, Institute of Molecular Biosciences, Mahidol University, Nakhon Pathom, Thailand; 2 Department of Microbiology, Faculty of Medicine Siriraj Hospital, Mahidol University, Bangkok, Thailand

**Keywords:** antigenic diversity, dengue, DENV3, intra-serotypic, Thailand

## Abstract

In addition to the well-known differences among the four dengue serotypes, intra-serotypic antigenic diversity has been proposed to play a role in viral evolution and epidemic fluctuation. A replacement of genotype II by genotype III of dengue virus serotype 3 (DENV3) occurred in Thailand during 2007–2014, raising questions about the role of intra-serotypic antigenic differences in this genotype shift. We characterized the antigenic difference of DENV3 of genotypes II and III in Thailand, utilizing a neutralizing antibody assay with DENV3 vaccine sera and monotypic DENV3 sera. Although there was significant antigenic diversity among the DENV3, it did not clearly associate with the genotype. Our data therefore do not support the role of intra-serotypic antigenic difference in the genotype replacement. Amino acid alignment showed that eight positions are potentially associated with diversity between distinct antigenic subgroups. Most of these amino acids were found in envelope domain II. Some positions (aa81, aa124, and aa172) were located on the surface of virus particles, probably involving the neutralization sensitivity. Notably, the strains of both genotypes II and III showed clear antigenic differences from the vaccine genotype I strain. Whether this differencewill affect vaccine efficacy requires further studies.

## Introduction

Dengue viruses are classified into four serotypes, which are believed to evolve to maintain equi-antigenic distances from one another. The equidistance optimizes antibody-dependent enhancement and facilitates epidemic spread. Primary infection by one serotype is believed to result in protection against subsequent infection by the same serotype. Strains within the same serotype are often considered similar in antigenicity. However, it was previously shown that substantial antigenic diversity existed within serotype [1]. The intra-serotypic antigenic diversity could result in reinfection by a different strain of the same serotype [2]. Although the role of intra-serotypic antigenic diversity is still unclear, it was proposed that an increased intra-serotypic antigenic distance may contribute to epidemic [1, 3]. Whether this was because of more frequent reinfection of the same serotype or an increased effectiveness of antibody-dependent enhancement is unclear.

In a large-scale analysis of dengue evolution in Bangkok, Thailand, covering two decades of 1994–2014, the antigenic diversity of dengue viruses did not always correlate with the serotype [3]. Some strains may be antigenically closer to strains of different serotypes than to some strains of the same serotype. In a long-term trend, serotypes appeared to evolve to increase antigenic distances from one another, whereas intra-serotypic antigenic diversity resonated to increase the distance over a period and then decrease in the following period. This resonance limited the overall long-term intra-serotypic antigenic diversity. During the period of this antigenic resonance, an increase in intra-serotypic antigenic distance and a decrease in inter-serotypic distance coincided with larger epidemic of that serotype. This suggested that reinfection and replacement of strains with novel antigenic property evading existing immunity against that serotype may contribute to the epidemic spread. On the other hand, the changes in inter- and intra-serotypic antigenic distance may have made antibody-dependent enhancement more optimal and hence contributed to the epidemic spread.

Each dengue serotype is composed of multiple genotypes, which may co-circulate in a geographic region or distribute differently in different regions. Dengue virus serotype 3 (DENV3) comprises four genotypes: genotype I (Indonesia, Malaysia, and South Pacific Ocean Island), genotype II (Thailand and Bangladesh), genotype III (Sri Lanka and India), and genotype IV (Latin America and Central America) [4].

In Thailand, there was a genotype displacement, in which genotype III replaced genotype II [3]. There was no clear increase in the epidemic of DENV3 during this genotype replacement period. It was suggested that the genotype replacement was caused by an immune-selective pressure from different serotypes. In order to gain more insight into the intra-serotypic antigenic diversity and its role in viral evolution, we characterized the antigenic diversity of DENV3 in both genotypes II and III from Thailand, covering strains from 2004 to 2015.

## Materials and methods

### Cells, viruses, and sera

African green monkey kidney (Vero) cells were grown in Minimum Essential Medium (MEM; Gibco) with 10% fetal bovine serum (FBS; Gibco), 100 Units/ml of penicillin, and 100 μg/ml of streptomycin (Millipore) and maintained in 5%CO_2_ at 37°C. Mosquito C6/36 cells were cultured in Leibovitz’s L-15 Medium (HyClone) supplemented with 5% FBS and 3% tryptose phosphate broth. C6/36 cells were maintained at 28°C without CO_2_. The use of anonymized human samples was approved by the Mahidol University Central Institutional Review Board (MU-CIRB) (COE No. MU-CIRB 2023/117.0707). The collection of monotypic human dengue sera from adult volunteers and dengue vaccinees who were immunized with live-attenuated DENV3 strain 16562 candidate vaccine was used in this study.

DENV3 strains were isolated from samples of dengue patients during epidemic in Thailand during 2004–2015. Laboratory strains of DENVs (DENV1 strain 16007, DENV2 strain 16681, DENV3 strain 16562, and DENV4 strain 1036) and Japanese encephalitis virus (JEV) strain Beijing were used for screening monotypic sera in this study. Viruses were propagated in C6/36 and Vero cells with MEM containing 2% FBS. Virus culture fluid was harvested when the cells reached full cytopathic effect. The virus titre was determined by the focus formation assay.

### Sequence analysis of dengue envelope gene

Dengue viral ribonucleic acid (RNA) was isolated from virus stock using phenol–chloroform extraction (TRIzol). The extracted RNA was reversely transcribed into complementary deoxyribonucleic acid (cDNA) by SuperScript® III reverse transcriptase (Invitrogen) by random hexamer primer according to the manufacturer’s protocol. Then, polymerase chain reactions (PCRs) were performed by high-fidelity Phusion Taq Polymerase (Thermo Fisher Scientific) with dengue 1–4 specific forward primers (5’ GAYRTWGAYTGYTGGTGYAA 3′) and dengue 1–4 reverse primers (5’ AAYTTRTAYTGYTCTGTCAA 3′). The reactions included the following: 30 s at 98 °C, followed by 35 cycles for 10 s at 98 °C, 30 s at 55 °C, 2 min at 72 °C, and 1 cycle for 7 min at 72 °C. The PCR products were subjected to 1% agarose gel electrophoresis and purified by the QIAquick Gel Extraction Kit (Qiagen). The purified PCR products were sent for sequencing to Macrogen (Macrogen, Seoul, Korea).

For phylogenetic tree analysis and genotyping, the 61 dengue envelope nucleotide and amino acid sequences were collected from the National Center for Biotechnology Information (NCBI) and aligned using ClustalW multiple alignment in BioEdit [5]. The phylogenetic tree was analysed using MEGA version 7.0.26 [6].

### Focus reduction neutralization test (FRNT)

For neutralization assay, sera were inactivated at 56°C for 30 min. Briefly, Vero cells were seeded in 24-well plates at 4 × 10^5^ cells/well. The serum samples were twofold serially diluted by MEM (Gibco, NY, USA) containing FBS (HyClone Laboratories, Pasching, Austria). Each dilution was then mixed with dengue virus at 50 focus-forming units (FFUs)/well for 60 min. The virus–serum mixture was infected into Vero monolayer cells for 90 min at 37 °C and 5% CO_2_. Following infection, cells were overlaid with semisolid MEM containing 5% FBS and 2% carboxymethyl cellulose. Cells were fixed with 80% acetone solution for 5 min and washed with PBS-Tween 20. Cells were incubated with anti-flavivirus 4G2 monoclonal antibody at 37 °C for 60 min. After washing with PBS-T, cells were incubated with polyclonal goat anti-mouse IgG conjugated with alkaline phosphatase (Jackson ImmunoResearch) at 37 °C for 60 min. Then, cells were washed two times with PBS-T before adding 5-bromo-4-chloro-3-indolyl phosphate/nitro blue tetrazolium (BCIP/NBT) substrate (Sigma, MO, USA) to visualize the foci of dengue virus. Foci were counted. Data were calculated by probit analysis using Statistical Package for the Social Sciences (SPSS) version 16.0 program as FRNT50 titre, which is a reciprocal dilution of serum that neutralizes 50% of virus input. The FRNT50 titre of ≥10 was considered as positive. The geometric mean titres (GMTs) with 95% confidence interval (CI) of the neutralizing antibody titres were calculated to determine the level of antibody titres of sera against each DENV.

### Antigenic map and molecular visualization

The neutralizing antibody titres of sera against individual DENV3 strains were analysed and visualized using an antigenic map [7]. The antigenic distance between grids indicates the difference in twofold neutralizing antibody titre. The crystal structure of DENV3 envelope homodimer from Protein Data Bank (PDB) is as follows: 7A3S was selected to visualize the potential amino acids using PyMOL software [8].

### Production of anti-DENV3 in monkey

The experimental protocol was approved by the Animal Care and Use Committee of the National Primate Research Center of Thailand, Chulalongkorn University (Protocol Review No. 1875005). A total of six healthy flavivirus-naïve Cynomolgus macaques (*Macaca fascicularis*) that were negative for anti-DENV 1–4 and anti-JEV were randomly assigned into two groups. Monkeys were subcutaneously injected with 10^5^ FFU of DENV3 isolates DENV3/TH/189/2006 and DENV3/TH/242/2015 on the upper arm at day 0. Clinical signs including food consumption were observed daily. Physical examination including body weight and body temperature was measured at the time of blood collection. The blood of injected animals was collected under anaesthesia on days 30, 60, and 90 after injection. The sera were prepared and used to determine the neutralizing activity by FRNT as mentioned above.

### Analysis of selection pressure in DENV3

DENV3 envelope gene sequences were obtained from the NCBI database and aligned using ClustalW. Gaps and ambiguities were removed appropriately. A total of 60 sequences, selected to represent the DENV3 genotypic diversity, were used as the dataset for the analysis. Phylogenetic analysis was performed using the Phylogenetic Analysis Using Parsimony (PAUP*) software package [9] with the ModelblockPAUPb10. Modeltest 3.7 [10] was employed to assess and select the most appropriate substitution model for the subsequent maximum-likelihood (ML) analysis. The ML analysis was conducted by utilizing the output from Modeltest 3.7 for ML search in PAUP*. The unrooted tree obtained was converted to Nexus format for further analysis. Codeml program, within the Phylogenetic Analysis by Maximum Likelihood (PAML) package, was used to estimate key parameters associated with molecular evolution [11]. The M0 model in Codeml was initially applied to estimate the nucleotide substitution rates (kappa) and the nonsynonymous-to-synonymous rate ratio (omega, ω). Subsequent analyses, including M1 (neutral), M2 (selection), M3 (discrete), M7 (beta), and M8 (beta and omega), were conducted to identify regions under positive selection. Interpretation of Codeml results involved assessing the likelihood of different evolutionary scenarios. Likelihood ratio tests (LRTs) were performed to compare nested models, such as M1 vs. M2, M1 vs. M3, and M7 vs. M8, using the chi-squared distribution, and P-values were calculated to determine the significance of model comparisons. A significant LRT result would indicate the models with a significantly better fit to the data than the corresponding neutral model (M1 or M7), implying the presence of positively selected sites.

## Results

### A genotype displacement of DENV3 from genotype II to genotype III in Thailand

**Figure 1.**
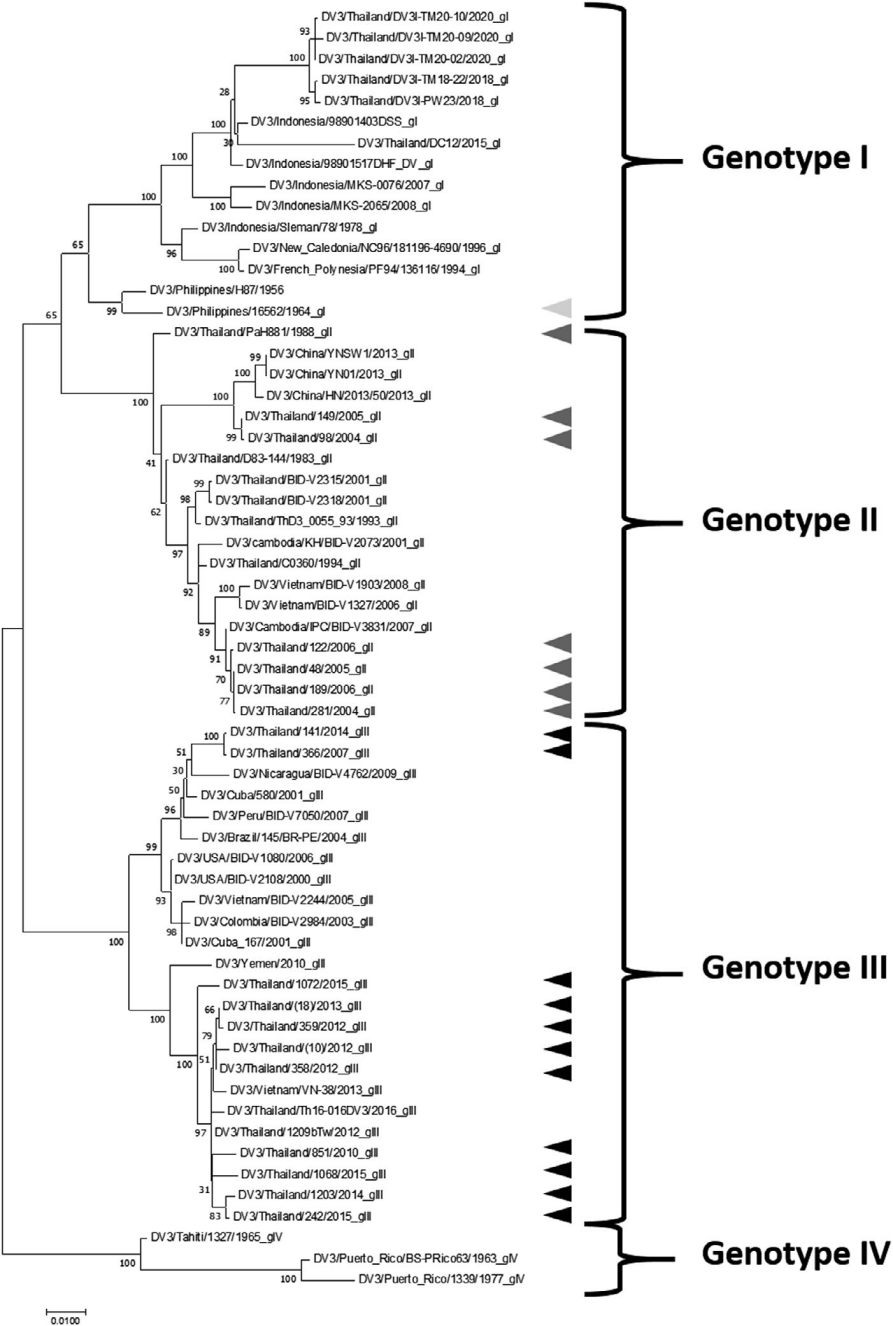
Phylogenetic tree analysis of DENV3 envelope nucleotide sequences using the maximum-likelihood method with 1,000 bootstrap replicates. The phylogeny was reconstructed using MEGA7 [6]. The arrowhead showed the dengue viruses used in this study. The data indicate DENV3/country/strain/year.

To investigate the intra-serotypic antigenic diversity, the DENV3 viruses isolated from human dengue patient during 2004–2015 in Thailand and two laboratory DENV3 strains 16562 and PaH881 were used in this study (Table 1). The analysis of DENV3 envelope sequences and the phylogenetic tree revealed that viruses isolated in Thailand during 1988–2006 were genotype II, whereas viruses isolated in 2006–2015 were genotype III (Figure 1). From the data, we found the replacement of genotype III with genotype II occurred in Thailand during 2006. Analysis of amino acid variation within the same genotype revealed a minor range of variation, between 1.11 and 2.37%. When analysing the amino acid variation between genotypes, a broader range of variation was observed, ranging from 2.42 to 5.00%.

**Table 1.** Dengue viruses used in this study

Viruses	Year	Country	Genotype
DENV3/PH/16562/1964	1964	Philippines	GI
DENV3/TH/PaH881/1988	1988	Thailand	GII
DENV3/TH/98/2004	2004	Thailand	GII
DENV3/TH/48/2005	2005	Thailand	GII
DENV3/TH/149/2005	2005	Thailand	GII
DENV3/TH/122/2006	2006	Thailand	GII
DENV3/TH/189/2006	2006	Thailand	GII
DENV3/TH/366/2007	2007	Thailand	GIII
DENV3/TH/851/2010	2010	Thailand	GIII
DENV3/TH/(10)/2012	2012	Thailand	GIII
DENV3/TH/358/2012	2012	Thailand	GIII
DENV3/TH/359/2012	2012	Thailand	GIII
DENV3/TH/(18)/2013	2013	Thailand	GIII
DENV3/TH/141/2014	2014	Thailand	GIII
DENV3/TH/1203/2014	2014	Thailand	GIII
DENV3/TH/242/2015	2015	Thailand	GIII
DENV3/TH/1068/2015	2015	Thailand	GIII
DENV3/TH/1072/2015	2015	Thailand	GIII

### The antigenic diversity of dengue virus 3 isolated in Thailand

**Figure 2.**
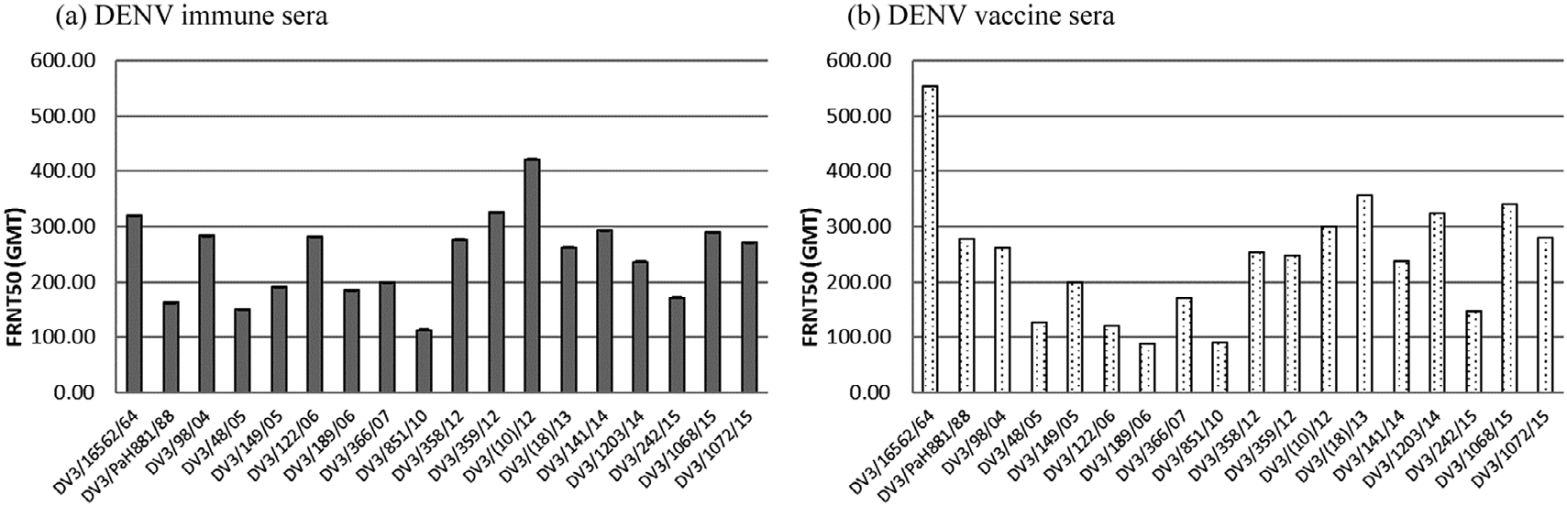
Average neutralizing antibody titers of (a) dengue immune sera and (b) dengue vaccine sera against individual DENV3.

To investigate the antigenic diversity of different genotypes of DENV3, the eighteen DENV3 isolates consisting of genotypes I, II, and III were tested for sensitivity to be neutralized by human dengue sera. The human sera used in this study were monotypic, having a positive antibody titre specifically to the DENV3 laboratory strain 16562, while exhibiting negative antibodies to other serotypes such as dengue 1 strain 16007, dengue 2 strain 16681, and dengue 4 strain 1036 and Japanese encephalitis. The human serum collection included DENV3 immune sera (s06/297, s07/327A, and s07/327B) and DENV3 vaccine sera obtained from individuals who have been vaccinated with the live-attenuated DENV3 strain 16562 (s06/293, s06/296, s08/256, s08/253, s06/97, s06/109). In general, it is believed that the sensitivity of dengue virus to be neutralized by dengue immune sera is not different within the same serotype. In this study, we found that all DENV3 can be neutralized by human dengue sera; however, with different levels of antibody titre. Some viruses such as DENV3/16562/64 and DENV3/TH/(10)/2012 exhibited high neutralization antibody titre, whereas some viruses such as DENV3/TH/851/2010 exhibited low neutralization antibody titre. The trend of level of neutralization antibody titre was not different between dengue immune sera and vaccine sera (Figure 2)

### Antigenic map revealed the group of antigenic diversity of dengue is not correlated with genotype of DENV3

**Figure 3.**
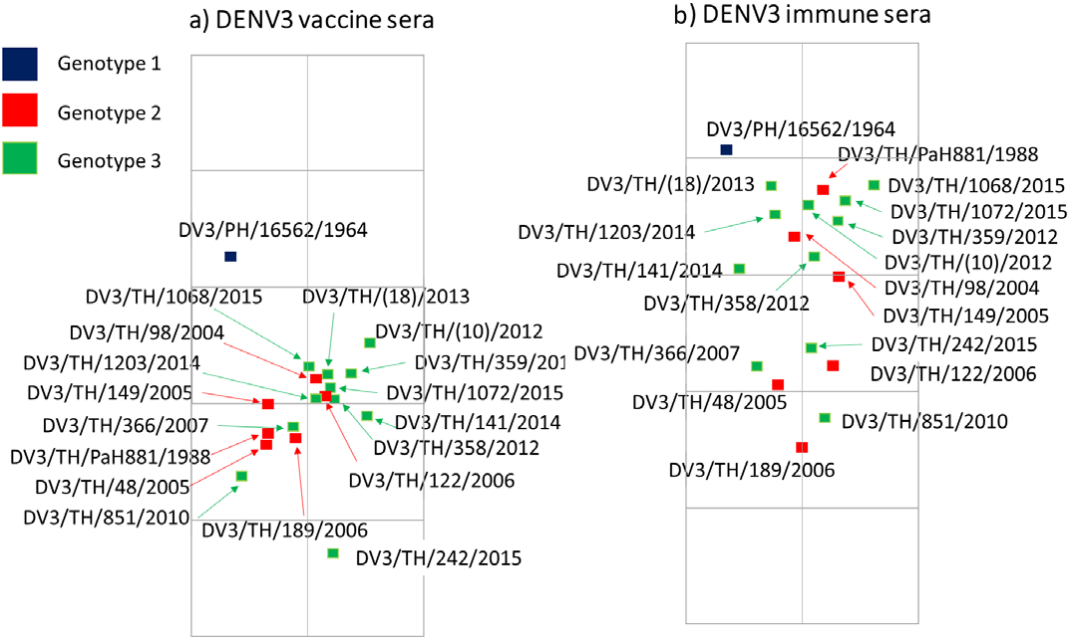
Antigenic maps were generated to visualize the antigenic characteristics of all genotypes of DENV3 using two sets of sera: (a) dengue vaccine sera and (b) dengue immune sera. The grid in the background scales to a twofold dilution of antisera.

To visualize the relation or difference of the antigenic diversity within DENV3, the neutralizing antibody titre was used to construct the antigenic map. The antigenic distance indicates the difference in average titre. The viruses exhibit a closely comparable antibody titre, revealing their close proximity on the antigenic map. As expected, dengue vaccine sera from immunization with DENV3 live attenuated vaccine (LAV)-derived strain 16562, which has a relatively high antibody titre to homologous strain, exhibited the antigenic map far distance from other genotypes. Interestingly, the viruses were prone to dividing into two subgroups. These subgroups did not exhibit a clear differentiation based on genotype, as both genotypes II and III are found within each group (Figure 3).

### Analysis of DENV3 amino acid sequences reveals potential amino acids contributing to antigenic variation

**Figure 4.**
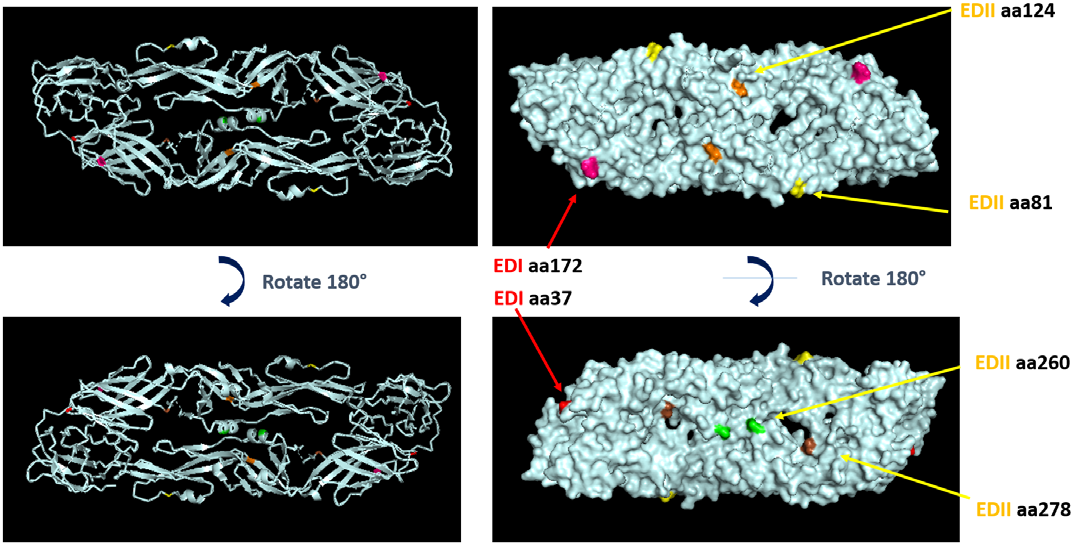
Crystal structure of DENV3 envelope homodimer PDB:7A3S was used to visualize the localization of significant amino acids by PyMOL software [8]. Upper is the outer surface, and lower indicates the inner surface, which rotates by 180 degrees. Each amino acid position displays in colours: aa81 (yellow), aa124 (orange), aa172 (pink), aa37 (red), aa260 (green), aa278 (brown).

The results from antigenic map of DENV3 exhibited the distinct separation into two subgroups. To further investigate, we examined the variation of amino acid sequences of envelope protein in each subgroup. The envelope protein of dengue virus is of particular interest as it serves as the main target for neutralizing antibodies. It comprises three domains: EDI, EDII, and EDIII. EDI and EDII are discontinuous peptides connected by four-peptide linker to form EDI/EDII hinge, while EDIII is a continuous peptide extended from EDI, which is immunoglobulin-like fold. Most of the viruses were grouped together, exhibiting similarities to those found in both the dengue vaccines and immune plots. However, there were viruses that could not be assigned to a specific subgroup due to their distinct grouping patterns, which differed from both the dengue vaccines and dengue immune plots. Consequently, the following viruses were excluded from our analysis: DENV3/TH/141/2014, DENV3/TH/122/2006, DENV3/TH/PaH881/1988, and DENV3/TH/149/2005. To determine the variation, the envelope amino acid sequences of each subgroup were compared. Subgroup 1 contained DENV3/TH/(10)/2012, DENV3/TH/(18)/2013, DENV3/TH/1068/2015, DENV3/TH/98/2004, DENV3/TH/1203/2014, DENV3/TH/358/2012, DENV3/TH/359/2012, and DENV3/TH/1072/2015. Subgroup 2 included DENV3/TH/48/2005, DENV3/TH/189/2006, DENV3/TH/242/2015, DENV3/TH/366/2007, and DENV3/TH/851/2010. The result showed that there were some distinct variations observed between each subgroup in each domain of envelope protein. These amino acid mutations appeared in the different majority of amino acids found in each subgroup or presented unique mutations not present in the other group. We identified the difference in eight amino acid positions in each subgroup (Table 2). Most of the significant amino acid positions were observed in EDII including aa81, aa124, aa260, aa271, and aa278. The graphic visualization of homodimer DENV3 envelope protein demonstrated that amino acid positions aa81, aa124, and aa172 were located on the surface of envelope protein. At other amino acid positions, aa37 was found on the side of envelope protein, whereas aa 260 and aa278 were located on the inner surface of DENV3 envelope protein (Figure 4). The importance of these amino acid positions involved in neutralizing needs to be further investigated.

Furthermore, to determine whether DENV3 genotypes differ in selection pressure, a total of 60 sequences representative of the DENV3 genotypic diversity from the NCBI programme were used as the dataset for the analysis. The Codeml program was applied to estimate the nucleotide substitution rates (kappa) and the nonsynonymous-to-synonymous rate ratio (omega, ω). Several models are used in codon-based analyses to understand the selective pressures acting on protein-coding genes. Model 8 showed the favoured model when using the LRT to compare models. The results provided two analysis parameters: Naive empirical Bayes (NEB) analysis typically provides point estimates without CIs or standard errors for the selection parameters, and Bayes empirical Bayes (BEB) provides more accurate estimates and is generally preferred for more reliable identification of positively selected sites. BEB analysis identifies sites with a high probability of positive selection, suggesting that these sites may have undergone adaptive evolution. A value of ω greater than 1 indicates positive selection, suggesting that nonsynonymous substitutions are favoured and may confer some adaptive advantages. In this study, the results showed ten amino acid residues obtained from M8, which was supported by a more favourable BEB analysis output (Table 3). Notably, position aa124 in the sequence demonstrated a significant likelihood of positive selection (Pr(ω >1) > 95%). In contrast, positions aa81, aa169, aa301, and aa479 displayed elevated probabilities, although they did not reach statistical significance. Positions aa81, aa124, aa260, and aa489 were identified to correspond with Table 2. Significant positive selection was found at position aa124, one of the potential determinants located on the surface. The next position with the most significant p value, although not reaching 95% significance level, was position aa81, which was also a potential determinant located on the surface of the protein. This made two of the three potential determinants located on the surface show evidence of positive selection. This further supports the role of these identified amino acid positions in the viral antigenic diversity and the role of antigenic changes in the viral evolution.

**Table 2.** Difference in amino acid variations identified in each subgroup

Envelope domain	aa position	subgroup1	subgroup 2	Variation	Location
EDI	37	N	N/S	*	Side
EDII	81	V/I	T/V/A	**	Outer surface
EDII	124	L/S	P/L	**	Outer surface
EDI	172	I	I/V	*	Outer surface
EDII	260	T/P	T/S	*	Inner surface
EDII	271	T/S	S/T	*	N/A
EDII	278	A/V	A	*	Inner surface
TM	489	V/T	A/V	*	N/A

*Note*: Envelope domain; EDI: aa1–52, aa133–191, aa279–294, EDII: aa53–132, aa192–278, EDIII: aa295–392, transmembrane (TM): aa393–493. aa. indicated the amino acid positions. * The different majority of amino acids found in each subgroup or present amino acids not found in another group. ** Distinct amino acid pattern observed within each subgroup. The majority of amino acids in each position displayed by the first alphabet.

**Table 3.** Analysis of the positively selected amino acid sites of the DENV3 envelope protein

Amino acid position	M8			
	NEB		BEB	
	Pr(ω >1)	ω	Pr(ω >1)	ω (±SEM)
81 I	0.699	1.365	0.911	1.427 ± 0.246
120 Q	–	–	0.551	1.008 ± 0.573
124 S	0.977*	1.723	0.983*	1.489 ± 0.105
132 H	–	–	0.507	0.954 ± 0.583
169 A	–	–	0.830	1.350 ± 0.346
260 T	–	–	0.597	1.120 ± 0.481
301 L	–	–	0.765	1.277 ± 0.419
380 I	–	–	0.523	1.018 ± 0.528
479 A	0.698	1.365	0.910	1.428 ± 0.242
489 A	–	–	0.555	1.054 ± 0.521

*Note*: Naive empirical Bayes (NEB) analysis typically provides point estimates without confidence intervals or standard errors for the selection parameters. Bayes empirical Bayes (BEB) analysis identifies sites with a high probability of positive selection, suggesting that these sites may have undergone adaptive evolution. A value of ω greater than 1 indicates positive selection.

* *P* value is >95%.

### Anti-DENV3 monkey sera exhibit concurrence of intra-serotypic variation observed in human dengue sera

**Figure 5.**
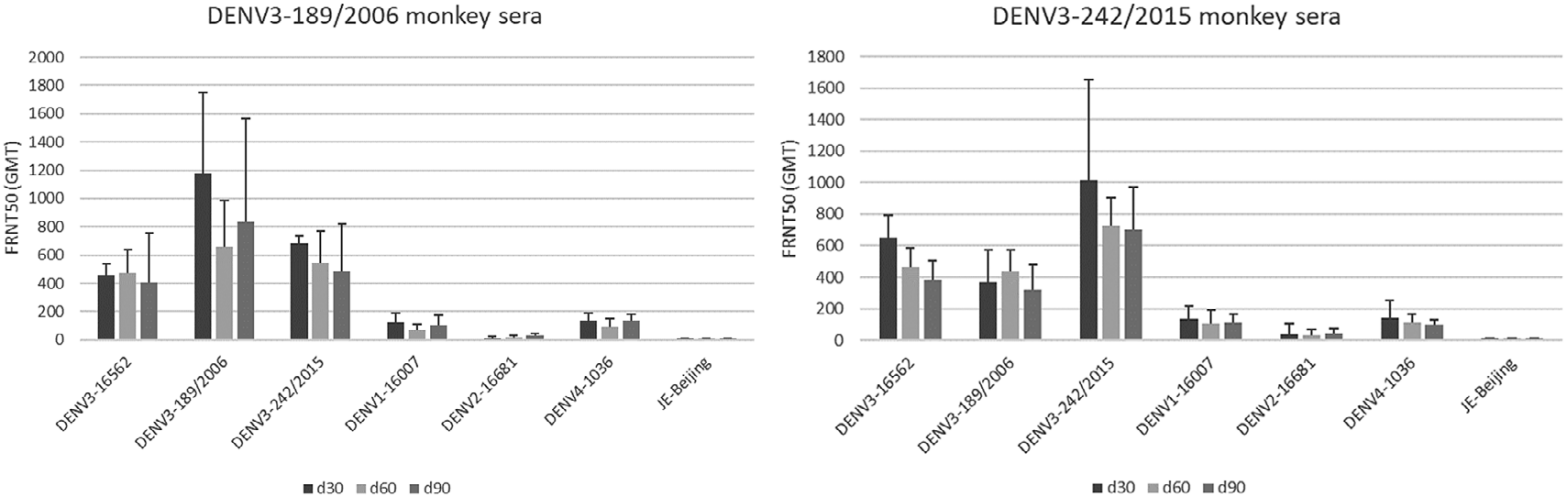
Neutralizing antibody titre of the sera from DENV3-injected cynomolgus monkeys to different genotypes of DENV3: DENV3/TH/189/2006 (genotype II) (left) and DENV3/TH/242/2015 (genotype III) (right). GMT; geometric mean titre, and error bars denoted 95% CI.

To demonstrate the characteristics of antibody responses from DENV3, two DENV3 isolates were selected. The chosen viruses were the DENV3 genotype II isolate DENV3/TH/189/2006 and genotype III isolate DENV3/TH/242/2015, which displayed the difference in antigenic distance by one block grid of antigenic map (approximately twofold dilution of neutralizing antibody titre). The flavivirus-naïve monkeys were subcutaneously injected with these viruses. The blood of the injected monkey was collected and used to determine the neutralizing antibody titres against different genotypes of DENV3 (genotypes I, II, and III); dengue 1, 2, and 4; and JEVs. The results demonstrated that both DENV3 infected monkeys elicited the highest neutralizing antibody titres against the homologous virus and were also capable of inducing such responses against heterologous genotypes of DENV3 (Figure 5). The kinetics of antibody responses varied between each virus, with the peak of antibody levels observed at day 30 for genotype 2 isolate DENV3/TH/189/2006 and at day 90 for genotype 3 isolate DENV3/TH/242/2015. In addition, cross-reactive antibodies to other dengue serotypes can be detected at low levels. No cross-reactive antibody was detected against JEV. Interestingly, the level of neutralizing antibody titre was approximately twofold difference correlated with the variation of antibody titre observed in human sera. Therefore, these findings provide support for the similarity of antigenic variations within the dengue serotype, as observed in human dengue sera, with those found in monkey sera infected with DENV3 viruses.

Furthermore, the amino acid sequences of DENV3, which were used in the production of anti-DENV3 monkey sera, were analysed to identify specific amino acid positions potentially associated with the antigenic diversity of DENV3. The analysis revealed that 15 amino acid positions differed between DENV3/TH/189/2006 and DENV3/TH/242/2015 strains. Among these amino acid variations, five mutations were identified as mutations potentially contributing to antigenic diversity (Table 4). Most mutations were observed in EDII. Consequently, it is likely that the antigenic variations observed in these viruses may be attributed to these significant mutations.

**Table 4.** Difference in amino acid positions of DENV3 used to produce anti-dengue monkey sera

Envelope domain	aa position	DENV3/TH/189/2006	DENV3/TH/242/2015	Variation
EDI	37	S	N	
EDII	81	T	V	**
EDII	93	K	E	
EDII	124	P	L	**
EDI	154	D	E	
EDI	160	V	A	
EDI	169	V	T	
EDI	172	V	I	*
EDII	271	S	T	*
EDIII	301	L	T	
EDIII	380	I	T	
EDIII	383	K	N	
EDIII	452	I	V	
EDIII	479	V	A	
EDIII	489	A	V	*

*Note*: Envelope domain; EDI: aa1–52, aa133–191, aa279–294, EDII: aa53–132, aa192–278, EDIII: aa295–392, transmembrane (TM): aa393–493. aa. indicated the amino acid positions. * The different majority of amino acids found in each subgroup or present amino acids not found in another group. ** Distinct amino acid pattern observed within each subgroup. The majority of amino acids in each position displayed by the first alphabet.

## Discussion

Although the antigenic diversity of DENV3 has been examined, the role of intra-serotypic diversity remains unclear. In this study, we aimed to investigate the antigenic diversity of DENV3 strains collected during the period of 2004–2015 in Thailand. Notably, the replacement of genotypes in dengue 3 from genotype II to genotype III has been previously documented in Thailand [3]. Consistent with these findings, all DENV3 isolates obtained from human patients in our study from 2007 to 2015 belonged to genotype III; whereas DENV3 genotypes isolated before 2006 belonged to genotype II. The underlying cause of the genotype replacement event remains largely unknown. It is hypothesized that changes in the antigenic diversity of DENV3 may be driven by natural selection or immune pressure [3]. However, our observations, based on the neutralization sensitivity of sera collected in 2006, did not reveal any significant differences between genotype II and genotype III. Moreover, the switch in genotypes may have been facilitated by the magnitude of DENV3 epidemics, particularly considering the rarity of DENV3 cases during 2005–2007, the years preceding the replacement event. Subsequently, genotype III of DENV3 became predominant [12, 13].

In our analysis, an antigenic map based on neutralizing antibody levels revealed the presence of subgroups within DENV3. Surprisingly, these subgroups did not seem to correlate with the genotypes observed in dengue vaccines and immune serum plots. The subgroups exhibited a separation of two- to four-fold antigenic distance. Within our study, we identified eight amino acid positions in the envelope protein that likely contribute to the antigenic diversity between these subgroups. Notably, the majority of these amino acids were found in the EDII region. Even though the DENV envelope protein does not participate in the replication of the viral RNA, it is essential for multiple stages of the virus lifecycle. Studies have reported that changes in the amino acid composition of the envelope proteins can result in an increase or decrease in their binding to cell receptors, subsequently affecting the infectivity of the virus particle. In a mature dengue virus particle, the envelopes lied flat antiparallel dimers with a fusion loop, consisting of three domains: a central β-barrel (EDI), an elongated finger-like dimerization region (EDII) that includes the fusion loop and is conserved among flaviviruses, and an exposed immunoglobulin-like β-barrel structure (EDIII) containing cellular-binding motifs. The C-terminus of EDIII has a stem region with two α-helices (EH1 and EH2) and a conserved sequence between them. This stem region also contains two transmembrane helix regions (TM1 and TM2), which retain the envelope protein in the endoplasmic reticulum. Certain key amino acids on the envelope protein have been identified to have an effect on cell attachment or viral entry [14]. Our results indicate that amino acid variations identified between subgroups could impact the neutralizing sensitivity of DENV3. Some of the amino acids (aa81, aa124, and aa172) were located on the outer surface of the virus particle, potentially affecting the sensitivity of neutralization. Besides, two of the three potential determinants (aa124 and aa81) located on the surface show evidence of positive selection. We found amino acid positions (aa169, aa301, and aa479) with a trend towards positive selection, albeit not reaching 95% significance level. These positions were not found among the potential determinants of the antigenic difference, suggesting that they did not directly contribute to the antigenic changes. However, they might still be positively selected and support antigenic site mutations as compensatory mutations, which suggests that the antigenic site mutations might affect the viral fitness and this trade-off effect might require compensation. To the best of our knowledge, there have been no reports regarding the importance of these specific amino acid residues. Further investigations are necessary to investigate their significance. We investigate the immune responses of monkeys injected by different DENV3 isolates. Our observations showed that the infection generated high antibody titres against the homotypic virus. However, we also observed a low level of cross-reactive antibodies to other serotypes. Due to the fact that DENV envelope protein contains conserved epitopes, it has the capacity to stimulate both complex and sub-complex neutralizing antibodies against other DENV serotypes [15]. These findings are supported by the similarity of DENV envelope amino acid sequences among DENV serotypes and JEV used in this study. Specifically, DENV3 (DENV3 16562, DENV3/TH/189/2006, and DENV3/TH/242/2015) shared 96.5% similarity, whereas DENV3 to other DENV serotypes (DENV1–16007, DENV2–16681, and DENV4–1036) shared 77.7–78.3%, 67.4–68.4%, and 63.0–63.2%, respectively. On the other hand, no cross-reactivity was found with JEV, which was related to the percentage of similarity of envelope amino acid sequences between DENV3 and JEV, which ranged from 47.3% to 47.9%.

The development of dengue vaccine should concern about the stimulation of broad protective antibody responses against all serotypes and genotypes. In our study, we have demonstrated that sera from individuals vaccinated with a live-attenuated DENV3 vaccine strain belonging to genotype I were able to neutralize the majority of DENV3 genotypes.

However, it is important to note that our study had some limitations regarding the virus strains used. Specifically, genotypes IV was not observed, and the presence of genotype I was very rare in Thailand [16]. These limitations should be taken into account when interpreting our finding. Therefore, our analysis of antigenic diversity was primarily focused on genotypes II and III, along with a single strain of genotype I. Through sequence analysis of the envelope protein, we observed that the DENV3 strain 16562 of genotype I differed by approximately 3.1% and 3.7% from genotypes II and III, respectively. Furthermore, the antigenic distance of genotype I appeared to be considerable when compared to other genotypes. It remains to be investigated whether these differences have any impact on the overall neutralizing sensitivity.

## Conclusion

The present study showed the antigenic diversity within the serotype of DENV3 isolated from DENV3 Thai patients. Sequence analysis of DENV3 revealed multiple genotypes. However, the antigenic diversity, determined by neutralizing antibody titre, did not demonstrate clearly association with the genotype. We highlighted that amino acid positions differed between distinct antigenic subgroups are potentially associated with antigenic diversity. Most of the potential amino acid determinants were found in EDII. Some of these amino acids are located on the surface of virus particle, probably contributing to the sensitivity to be neutralized by sera. Therefore, our data do not support the role of intra-serotypic antigenic variation. Since the development of vaccine concerns broad protection of antibody responses, further studies are necessary to investigate the impact of these differences on vaccine efficacy.

## Data Availability

All the evidence and data generated and analysed in this research finding will be available to readers without undue barriers by the corresponding author upon reasonable request.
